# Reduced Albuminuria and Potassemia Indicate Early Renal Repair Processes after Resynchronization Therapy in Cardiorenal Syndrome Type 2

**DOI:** 10.1155/2020/2727108

**Published:** 2020-03-21

**Authors:** Agnieszka Gala-Błądzińska, Janusz Romanek, Danuta Mazur, Tomasz Stepek, Marcin Braun, Piotr Szafarz, Marcin Chlebuś, Andrzej Przybylski

**Affiliations:** ^1^Medical College of Rzeszów University, Institute of Medical Sciences, Rzeszów 35-310, Poland; ^2^Department of Internal Medicine, Nephrology and Endocrinology, St. Queen Jadwiga Clinical District Hospital No. 2 in Rzeszów, Rzeszów 35-301, Poland; ^3^Department of Cardiology, St. Queen Jadwiga Clinical District Hospital No. 2 in Rzeszów, Rzeszów 35-301, Poland; ^4^Department of Pathology, Chair of Oncology, Medical University of Lodz, Łódź 90-647, Poland

## Abstract

**Background:**

Patients with chronic cardiorenal syndrome type 2 (T2-CRS) who qualify for resynchronization therapy (CRT) are exposed perioperatively to potentially nephrotoxic factors including contrast agents and blood loss.

**Methods:**

The objective of this prospective interventional study was to assess the effects of CRT on renal function in patients with T2-CRS within the first 48 hours following implantation. Initially, 76 patients (15% female; aged 69 ± 9.56 years) with heart failure (New York Heart Association classes II–IV), ejection fraction ≤ 35%, and QRS > 130 ms were included in the study. During CRT implantation, a nonionic contrast agent (72.2 ± 44.9 mL) was administered. Prior to and 48 hours following implantation, renal function was evaluated using the following serum biomarkers: creatinine (sCr), estimated glomerular filtration rate (using the Chronic Kidney Disease Epidemiology Collaboration equation [eGFR_CKD-EPI_]), and the electrolyte and urine biomarkers albumin (uAlb), albumin/creatinine ratio (UACR), and neutrophil gelatinase-associated lipocalin (uNGAL).

**Results:**

Before CRT, patients classified as NYHA class III or IV had higher uNGAL levels in comparison to uNGAL levels after CRT (43.63 ± 60.02 versus 16.63 ± 18.19; *p*=0.041). After CRT implantation, uAlb, UACR, and potassium levels were reduced (*p* < 0.05), and uNGAL, sCr, and eGFR_CKD-EPI_ were unchanged. The contrast medium volume did not correlate with the test biomarkers (*p* > 0.05).

**Conclusions:**

In patients with T2-CRS, uNGAL is a biomarker of kidney injury that correlates with the NYHA classes. A stable uNGAL value before and after CRT implantation confirms the lack of risk of contrast-induced nephropathy. Reduced albuminuria and blood potassium are biomarkers of improving T2-CRS in the early post-CRT period.

## 1. Introduction

Chronic kidney disease (CKD), abnormalities in kidney structure or function present for >3 months with health implications [1], affects 10 to 16% of all adults [2]. It is known that CKD significantly increases the risk for cardiovascular disease [3, 4]. In addition, chronic heart failure (CHF), affecting 1-2% of the population [5], often coexists with CKD. Both of these conditions drive the pathophysiology of cardiorenal syndrome (CRS). Large cohort studies have shown that chronic CRS is present in 45–63% of patients with CHF [6]. The coexistence of CHF and CKD worsens patient prognosis severalfold and increases the risk of death in this population and, in proportion, correlates with a decrease in the glomerular filtration rate [7, 8]. Studies of epidemiology and clinical experiences in nephrological and cardiological clinics have recommended the use of effective methods for the diagnosis and treatment of CRS to improve prognoses in the large patient populations. Resynchronization therapy (CRT) is a well-established treatment for patients with clinically apparent heart failure, decreased left ventricular ejection fraction (LVEF), and prolonged QRS duration on surface electrocardiogram (ECG) [9]. It is also known that CRT implantation reduces mortality in the CKD population overall [10]. Also, patients with renal dysfunction are poorly represented in CRT clinical trials, which results in a lack of evidence to guide treatment in this high-risk group [11]. Few studies have evaluated the impact of CRT implantation on kidney function, especially in the early postimplantation period in patients with chronic cardiorenal syndrome [12, 13]. In addition, the published studies evaluated only renal function using classic biomarkers such as serum creatinine, eGFR, and cystatin C, which do not accurately account for the pathomechanism of damage to renal structures. Meanwhile, CRT implantation accompanied by the use of contrast media may cause contrast-induced nephropathy (CIN) as a result of renal tubular damage.

The objective of this study was to assess the impact of CRT implantation on renal function as indicated by classic and modern biomarkers in patients with chronic cardiorenal syndrome type 2 (T2-CRS) within the first 48 hours following surgery.

## 2. Materials and Methods

**Figure 1:**
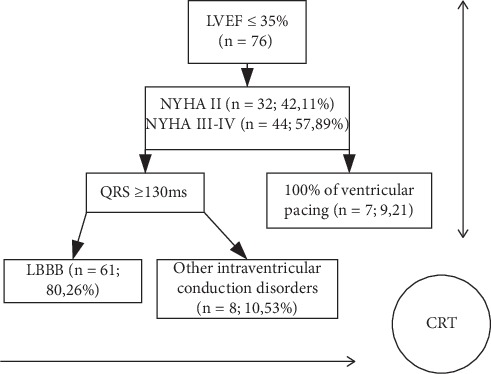
Algorithm for qualifying patients with chronic cardiorenal syndrome type 2, including indications for CRT implantation.

From September 1, 2016, until December 31, 2018, 76 patients with heart failure, who had met the criteria for CRT implantation according to the European Society of Cardiology guidelines for the treatment of heart failure, were prequalified for the prospective interventional study [14]. The algorithm for qualifying patients with T2-CRS for participation in the study, including indications for CRT implantation, is presented in Figure 1.

Nine (11.84%) patients had a resynchronization stimulator (CRT-P) implanted, and in all the other cases (*n* = 67; 88.16%), a procedure that included the option of defibrillator therapy (CRT-D) was used. CRT-D implantation was performed de novo in 50 (65.8%) people. In the CRT-D group, implantation of the device was primarily performed to prevent the risk of sudden death (*n* = 46; 60.52%) and to convert previous ventricular tachycardia/ventricular fibrillation arrhythmias (*n* = 4; 5.26%). In the remaining 17 (22.37%) patients, the previously implanted systems were upgraded. According to the recommendations of the European Society of Cardiology [14], CRT-P implantation was preferred in patients in NYHA class III or IV with more comorbidities and the absence of documented ventricular tachyarrhythmias.

The left ventricular electrodes were located in the lateral (*n* = 57; 75%), posterior-posterior (*n* = 5; 6.57%), and middle (*n* = 1; 1.35%) cardiac veins. Twelve patients (15.79%) were excluded from further analysis for the following reasons: presence of conditions causing an increase in uNGAL irrespective of kidney function (features of urinary tract infection in a general urine examination, focal change in the kidney, or thickening of the bladder mucosa; *n* = 6), failure to provide written consent or withdrawal of the patient's consent to participate in the study (*n* = 4), and left-cell electrode dislocation (*n* = 2).

Before implantation, each patient underwent a thorough physical examination with NYHA class assessment, cardiac echocardiography with LVEF, and abdominal ultrasound with urinary tract examination.

To determine cardiac function before CRT implantation, the NYHA heart rate class was assessed, an echocardiographic examination with LVEF was performed, and serum N-terminal pro-b-type natriuretic peptide levels were assessed (NT-proBNP).

To determine the effectiveness of CRT at 48 hours after implantation, the QRS width and NYHA heart rate class were assessed [15].

To assess renal function, the following classic biomarkers were used: serum creatinine (sCr), estimated glomerular filtration using the CKD-EPI equation (eGFR_CKD-EPI_), albuminuria in a single morning urine sample (uAlb), and the creatinine albumin ratio in a single morning urine sample (uACR). Additionally, the following modern biomarkers were assessed: lipocalin associated with neutrophil gelatinase in a single morning urine sample (uNGAL) and the uNGAL to creatinine ratio (uNCR) in a single morning urine sample.

Laboratory tests and the measurements of uNGAL, urine albumin, and urine creatinine were performed in the hospital laboratory. Urine NGAL concentrations were determined using the ARCHITECT® Analyzer (Abbott Core Laboratory, Abbott Park, IL, USA) and a chemiluminescent microparticle immunoassay.

Selected demographic and clinical data for participants and laboratory results prior to CRT implantation are presented in Table 1.

AKI was diagnosed according to the Kidney Disease Improving Global Outcomes (KDIGO) guidelines, that is, when serum creatinine increased to ≥26.5 mmol/L within 48 hours or ≥1.5 times, either verified or presumed to occur within 7 days [16].

During CRT implantation, a nonionic contrast agent (aqueous solution of iomeprol; osmolality of 726 ± 34 mOsm/kg of water) was administered. To assess the risk of contrast-induced nephropathy in the study population, the Mehran model [17] was used according to the following criteria: congestive heart failure: 5 points if NYHA class III/IV, or a history of pulmonary edema; age: 4 points if >75 years of age; anemia: 3 points if hematocrit <39% for men and <36% for women; diabetes: 3 points; volume of contrast medium: 1 point for every 100 ml; and eGFR in mL/min/1.73 m^2^: 2 points if 60–40, 4 points if 20–40, and 6 points if <20. Hypotonia requiring the use of pressure amines was not observed in the study group. No patient had an intra-aortic balloon pump.

All patients gave their written informed consent to participate in the study. The study was conducted in accordance with the Declaration of Helsinki and was approved by the Bioethics Committee of the University of Rzeszów (Reference No. 5/04/2016) N.

### 2.1. Statistical Analysis

Categorical variables are presented as numbers with percentages in brackets. Differences between categorical variables were evaluated using Pearson's chi-square test and Fisher's two-tailed exact test. Continuous variables are presented as medians with interquartile ranges in brackets. The Shapiro–Wilk test was used for the distribution assessment of continuous variables. Continuous variables with normal distribution were compared using Student's *t*-test for two groups, and one-way analysis of variance (ANOVA) with additional post hoc comparisons was used for three or more groups. Continuous variables with nonnormal distribution were compared using the Mann–Whitney *U* test for two groups or the Kruskal–Wallis ANOVA with additional post hoc comparisons for three or more groups. The Bonferroni correction for multiple testing was applied. Spearman's rank coefficient was calculated to assess correlations. The Statistica 12.5 PL package (Statsoft, Tulsa, OK, USA) was used for other analyses. A *p*value < 0.05 was considered statistically significant.

## 3. Results

Selected clinical and demographic data characterizing the study group prior to CRT implantation are described in Table 1.

To assess the initial renal function depending on the NYHA class, the research group was divided into two groups: NYHA II (*n* = 27) and NYHA III and IV combined (*n* = 37). For further statistical analysis, the NYHA classes III and IV were combined into one group, because in some patients it was difficult to clearly specify which of the two classes they belonged to, and because of the low number of subjects with evident heart failure among the NYHA class IV outpatients. Selected demographic and clinical data and test results in the study group prior to CRT implantation according to NYHA heart failure classes are described in Table 2.

**Figure 2:**
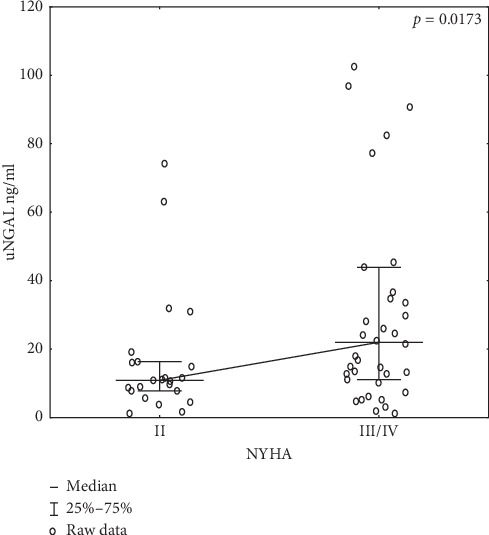
Graph showing the correlation of uNGAL with NYHA heart failure classes in the study group prior to CRT implantation; abbreviations: NYHA, New York Heart Association class; uNGAL, lipocalin associated with neutrophil gelatinase in a single sample of urine.

Significant correlations of uNGAL with NYHA heart failure classes in the study group prior to CRT implantation are presented in Figure 2.

The effect of concomitant T2DM or hypertension on renal function prior to CRT implantation was analyzed. No difference was observed in either group of patients prior to implantation: patients with T2DM (*p* > 0.05) and patients with hypertension (*p* > 0.05).

The effectiveness of CRT implantation was then assessed according to the study protocol. QRS complex narrowing was observed in all patients.

**Figure 3:**
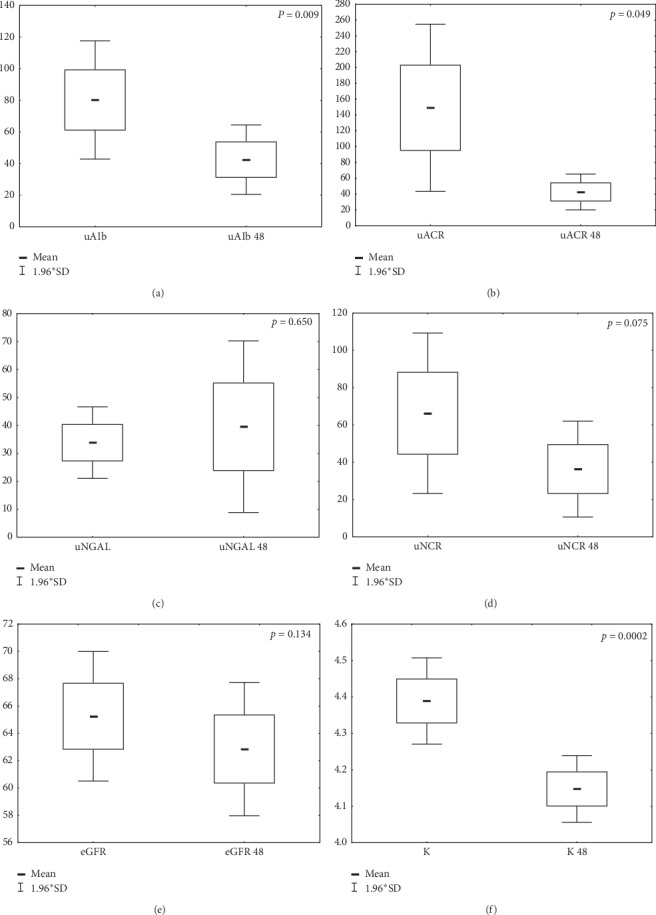
Impact of CRT implantation on selected renal parameters across the entire group of patients 48 hours following implantation. (a) uAlb, urinary albumin concentration in a single morning sample of urine prior to CRT implantation; uAlb 48, albumin concentration in a single morning sample of urine 48 hours following CRT implantation. (b) uACR, urinary albumin to creatinine ratio prior to CRT implantation; uACR 48, urinary albumin to creatinine ratio 48 hours following CRT implantation. (c) uNGAL, lipocalin associated with neutrophil gelatinase in a single morning sample of urine prior to CRT implantation; uNGAL 48, lipocalin associated with neutrophil gelatinase in a single morning sample of urine 48 hours following CRT implantation. (d) uNCR, urinary NGAL to creatinine ratio prior to CRT implantation; uNCR 48, urinary NGAL to creatinine ratio 48 hours following CRT implantation. (e) eGFR, estimated glomerular filtration rate calculated using Chronic Kidney Disease Epidemiology Collaboration prior to CRT implantation; eGFR 48, estimated glomerular filtration rate calculated using Chronic Kidney Disease Epidemiology Collaboration 48 hours following CRT implantation. (f) K, serum potassium concentration prior to CRT implantation; K 48, serum potassium concentration 48 hours following CRT implantation.

Recognizing that the CRT implantations were effective in most patients, the effect of CRT stimulation on patient renal function 48 hours following implantation was analyzed. The results are shown in Table 3 and Figure 3.

Selected laboratory parameters were evaluated in patients 48 hours following CRT implantation depending on the NYHA class. The relationships that were observed are presented in Table 4.

The impact of coexisting T2DM and HA on CRT implantation was also analyzed. It was observed that patients with T2DM had significantly lower albumin excretion (20.96 ± 29.16 mg/dL versus 66.40 ± 115.69 mg/dL; *p*=0.0317) and uACR (20.08 ± 32.88 mg/g versus 67.12 ± 115.73 mg/g; *p*=0.0314) 48 hours following CRT implantation compared with pre-CRT values, respectively. However, in the group of patients with concomitant HA, a significant decrease in eGFR was observed (60.81 ± 20.81 versus 65.06 ± 19.79; *p*=0.03) as well as a decrease in urinary albumin excretion (50.49 ± 98.82 versus 94.79 ± 164.97, *p*=0.02) 48 hours following CRT implantation compared with pre-CRT values, respectively.

During CRT implantation, a nonionic contrast agent (aqueous solution of iomeprol; osmolality of 726 ± 34 mOsm/kg of water) with an average dose of 72.2 ± 44.9 ml (median = 60 ml; quartiles: 50–100 mL) was administered. Using the Mehran risk score (MRS) [17], the risk of contrast-induced nephropathy was assessed in the studied population. Across that study's entire population, patients scored an average of 8.2 points (median 8 ± 4.38; min. 0; max. 19; quartiles: 2.66–14.11). In our study, there was no significant correlation between the MRS and the values of uNGAL and uNCR in patients 48 hours following CRT implantation (*p* > 0.05) [17]. Using the Spearman correlation, the effect of the volume of the contrast medium administered during CRT implantation on the value of the following biomarkers used in assessing renal function was determined: eGFR CKD-EPI, uNGAL, uNCR, uAlb, and uNCR as well as sodium, potassium, and blood morphotic values. No significant correlation between the tested biomarkers at 48 hours following implantation and the volume of the contrast medium was confirmed (*p* > 0.05).

Among the analyzed patients in whom CRT was implanted, an increase in creatinine above 0.3 mL/min from baseline was observed in two (3.125%) patients. According to the KDIGO 2012 definition [16], these patients were diagnosed with acute renal injury. The small size of this group does not allow for statistical analysis. None of the patients required renal replacement therapy.

## 4. Discussion

Significant comorbidities are common in patients with severe heart failure, as we have experienced in clinical practice. One of the conditions often associated with CHF is CKD. CKD resulting from CHF is known as T2-CRS [18, 19]. Patients with T2-CRS and symptomatic heart failure belong to a group at a high risk of mortality. At the same time, they are extremely vulnerable to the iatrogenic effects of the adverse effects of treatment. However, in long-term observations, effective treatment of heart failure usually results in improved kidney function and reduced mortality in this population [5, 19].

Therefore, we decided to assess the kidney function of patients with severe heart failure who qualified for CRT. Resynchronization is a method of cardiac pacing that aims to restore the optimal sequence of myocardial contraction and consequently improves both its systolic and diastolic functions, reduces functional mitral valve regurgitation, reverses adverse left ventricular remodeling, and reduces NYHA clinical symptoms [20].

Patients who met the T2-CRS criteria qualified for surgery in our study. In the study population, most patients met the classic CKD G1–G3/A1-A2 criteria according to KDIGO [1].

We observed a significantly larger increase in NGAL in a single sample of urine prior to CRT implantation in patients with heart failure in NYHA classes III and IV than in patients in NYHA class II. NGAL is a small protein with a molecular weight of 25-kDa, freely filtered through the glomeruli, and then reabsorbed into the blood in the proximal tubule [21]. The largest portion of NGAL in the urine is that fraction that results from the increased expression of NGAL mRNA in the thick ascending limb of the loop of Henle and collecting duct in response to a renal tubular damaging agent, such as a contrast agent [22, 23]. Urinary NGAL protein has been extensively studied in CRS and has both diagnostic and prognostic values in cases of heart failure with concomitant acute kidney damage [24]. The fact that higher NGAL values were found in our study in a single urine sample prior to CRT implantation in NYHA class III-IV patients than in NYHA class II patients indicated renal tubular damage in the NYHA III-IV group. Therefore, NGAL in a single urine sample may be one of the biomarkers for assessing renal function in T2-CRS that correlates with the clinical severity of CHF. In a large population study, Damman et al. observed that, in patients with CHF, renal interstitial damage was associated with an increase in uNGAL even with normal eGFR. Additionally, those researchers showed a positive correlation between uNGAL concentration and mortality and uNGAL and the number of hospitalizations in patients with CRS [25].

It should be remembered that the assessment of uNGAL as an indicator of renal function can only be objective after excluding factors that may affect its concentration regardless of renal function [14, 26]. Hence, our study excluded patients with systemic infection, features of urinary tract infection in a general urine test, suspected kidney cancer, and suspected bladder cancer.

Implantation patients are exposed to a contrast medium during the procedure. This factor may contribute to the possibility of deterioration of renal function in the perioperative period due to the prerenal mechanism (blood loss) as well as the renal-derived mechanism (contrast-induced nephropathy). In our study, at 48 hours following successful CRT implantation, we observed that all patients with T2-CRS, despite the use of a potentially nephrotoxic contrast agent and anemization, revealed a significant improvement in albuminuria and no increase in uNGAL. Hence, we believe that the assessment of albuminuria at 48 hours following CRT implantation provides a relatively immediate indicator of the effectiveness of implantation in terms of improved renal function in patients with T2-CRS. In addition, in our study, the largest decrease in albuminuria was observed in the group of patients with higher NYHA classes and coexisting T2DM. A decrease in albuminuria is a classic and important prognostic factor for improved renal function, reduced total mortality, reduced mortality due to cardiovascular reasons, and a reduction in the number of repeated hospitalizations in patients with heart failure [26–28]. It was observed in the Mangiavacchi et al.'s study that the implantation of CRT effectively prevented death and exacerbation of heart failure, both in the group of patients with T2DM and among those without diabetes [29]. Haase et al. [30] analyzed 10 prospective studies involving over 2,000 critically ill patients predominantly with a cardiorenal syndrome that examined the use of uNGAL in the diagnosis of AKI. Results showed that an increase in uNGAL is a biomarker for “subclinical AKI.” In addition, in patients after the percutaneous coronary intervention (PCI), an increase in uNGAL observed 48 hours following surgery is potentially useful in predicting a persistent increase in creatinine in contrast-induced AKI patients and may also allow the early identification of high-risk patients following PCI [31]. No increase in uNGAL observed 48 hours following CRT implantation in our study may suggest that no damage occurred to the intrasurgical renal tubules. In our study, we also observed that, regardless of its volume, the contrast agent administered during CRT implantation did not affect renal function as determined by all the biomarkers tested, including uNGAL, in the immediate postoperative period. In addition, the majority of patients in our study were characterized by comorbidities predisposing them to the occurrence of postcontrast nephropathy, including the following (apart from circulatory failure): age > 75 years, coexistence of anemia, diabetes, hypertension, and CKD with an eGFR < 60 mL/min/1.73 m^2^ [17]. Our observations showed that the MRS [17], which takes into account the comorbidities mentioned in the population of patients undergoing PCI to assess the risk of postcontrast nephropathy, does not correlate with the risk of postcontrast nephropathy in patients who qualified for CRT [32]. An interesting clinical observation that results from our study is the significant decrease in potassium in patients following CRT implantation regardless of the severity of the clinical symptoms of heart failure expressed by the NYHA scale as well as concomitant diseases. In patients with CKD and coexisting chronic CRS, the use of RAA-inhibiting drugs (RAAS-Is) is an important and recommended part of pharmacotherapy resulting from the pathophysiology of chronic CRS [14, 19]. Meanwhile, although RAAS-Is reduce mortality and morbidity in heart failure patients, clinicians must use RAAS-I drugs with caution because of the potential for life-threatening hyperkalemia. There are several pharmacological strategies to prevent this complication, but they are not always effective [33]. In the available literature, we have not found any reports on the effect of CRT on potassium in patients with T2-CRS. The decrease in serum potassium 48 hours following CRT implantation that was observed in our study probably results from subclinical improvement of the renal filtration function and presents an important function of CRT implantation in multifactorial T2-CRS treatment. From a large meta-analysis of published studies, it is known that the use of CRT in patients with CKD positively affects patient survival; however, the mechanism by which this occurs is unknown. The authors of our study strongly recommend that a randomized controlled trial should be conducted to define the role of CRT implantation in patients with CKD [10].

Despite some interesting observations and results, in our opinion, the current research work has its limitations. The limitations of the study are the small size of the research group and the lack of a control group.

## 5. Conclusions

uNGAL is a biomarker of kidney function that correlates with NYHA classes in T2-CRS patients. CRT implantation in patients with T2-CRS is not a risk factor for postcontrast nephropathy as evidenced by the lack of uNGAL and uNCR increases and stable glomerular filtration in patients 48 hours following CRT implantation.

Assessment of albuminuria 48 hours following CRT implantation can be considered an early and noninvasive biomarker of implantation efficiency in terms of improved renal function in patients with T2-CRS. This finding is clinically relevant considering the proven association of increased albuminuria with the risk of cardiovascular complications and the progression of CKD. A significant decrease in potassium levels 48 hours following the implantation of CRT is a biomarker of subclinical improvement in renal function in patients with T2-CRS, regardless of the severity of any clinical symptoms of heart failure or comorbidities. [31–33]

## Figures and Tables

**Table 1:** Selected demographic and clinical data and laboratory results of the study group prior to CRT implantation (Mann–Whitney *U* test with continuity correction).

Variable	CHF (*n* = 76)		
	Median	Lower quartile	Upper quartile
Age, years	69	62.5	76
Female gender, *n* (%)	18 (23.68)		
BMI, kg/m^2^	28.2	25.4	31.8
Etiology of CHF, *n* (%)			
CAD	49 (64.1)		
DCM	23 (31.2)		
Other	4 (4.7)		
LVEF (%)	25	20	30
NT-proBNP, pg/ml	2916	1164	5918
NYHA II, *n* (%)	32 (42.11)		
NYHA III, III/IV, or IV, *n* (%)	44 (57.89)		
Intraventricular conduction disorders, *n* (%)			
LBBB	59 (77.63)		
100% percentage of right ventricular pacing	17 (22.37)		
Chronic AF	26 (34.38)		
Paroxysmal AF	18 (23.44)		
QRS width, ms	160	147	187
CKD stadiums, *n* (%)			
G1-G2	43 (56.58)		
G3	30 (39.47)		
G4	3 (3.95)		
A1	48 (63.16)		
A2	4 (5.26)		
A3	24 (31.58)		
Total cholesterol (mg/dl)	171	144	200
Cholesterol LDL (mg/dl)	94	78	117
Cholesterol HDL (mg/dl)	42	35	49
TG (mg/dl)	131	94	168
T2DM, *n* (%)	31 (48.44)		
HA, *n* (%)	49 (76.56)		
RAAS-Is used, *n* (%)	76 (100)		
Statin used, *n* (%)	76 (100)		

Abbreviations: BMI, body mass index; CHF, chronic heart failure; CKD, chronic kidney disease stadium by KDIGO 2012 [1]; CAD, coronary artery disease, DCM, dilated cardiomyopathy; LVEF, left ventricular ejection fraction; NT-proBNP, *N*-terminal pro-b-type natriuretic peptide levels were assessed; NYHA, New York Heart Association class; LBBB, left bundle branch block; RBBB, right bundle branch block; AF, atrial fibrillation; LDL, low-density lipoprotein; HDL, high-density lipoprotein; TG, triglycerides; T2DM, type 2 diabetes; HA, hypertension; RAAS-Is, renin-angiotensin-aldosterone system inhibiting drugs.

**Table 2:** Selected demographic and clinical data and test results in the study group prior to CRT implantation according to NYHA heart failure classes.

Variable	NYHA II (*n* = 27)	NYHA III and IV (*n* = 37)	*p*
Age, years	67.75 ± 6.07	69.17 ± 10.5	0.547
Female gender, *n* (%)	2 (8.33)	13 (33.33)	0.033
BMI (kg/m^2^)	29.3 ± 5.48	28.8 ± 5.31	0.723
CAD, *n* (%)	18 (66.67)	29 (78.38)	0.071
T2DM, *n* (%)	15 (62.5)	16 (41.03)	0.097
HA, *n* (%)	21 (87.5)	28 (71.79)	0.214
sCr (mg/dL)	1.125 ± 0.35	1.19 ± 0.36	0.08
eGFR _CKD-EPI_ (mL/min/1,73 m^2^)	63.95 ± 19.15	63.04 ± 19.84	0.429
uNGAL (ng/mL)	16.63 ± 18.19	43.63 ± 60.02	0.041
uNCR (*µ*g/g)	53.73 ± 177.0	75.49 ± 155.08	0.056
uAlb (mg/dL)	92.32 ± 164.94	81.87 ± 138.65	0.079
uACR (mg/g)	207.42 ± 573.85	116.76 ± 231.52	0.291
uCr (mg/dL)	130.46 ± 80.83	97.82 ± 63.47	0.304
HCT (%)	41.58 ± 4.03	39.89 ± 4.5	0.029
Hb (g/dL)	13.73 ± 1.36	13.17 ± 1.79	0.214
RBC, millions per K/*µ*L	4.56 ± 0.42	4.50 ± 0.58	0.113
WBC, thousands per K/*µ*L	7.30 ± 2.22	7.28 ± 2.72	0.025
PK, thousands per K/*µ*L	197.5 ± 43.07	213.31 ± 69.97	0.114
Na (mmol/L)	137.08 ± 2.18	136.44 ± 3.44	0.085
K (mmol/L)	4.41 ± 0.31	4.38 ± 0.51	0.222

Abbreviations: BMI, body mass index; CAD, coronary artery disease; T2DM, type 2 diabetes; HA, hypertension; sCr, creatinine serum; eGFR CKD-EPI, estimated glomerular filtration rate calculated using Chronic Kidney Disease Epidemiology Collaboration; uNGAL, lipocalin associated with neutrophil gelatinase in a single sample of urine; uNCR, urinary NGAL to creatinine ratio; uAlb, urinary albumin concentration in a single morning sample of urine; uACR, urinary albumin to creatinine ratio; uCr, urea creatinine; HCT, hematocrit, Hb, hemoglobin; RBC, red blood cell count; WBC, white blood cell count; PK, platelet count; Na, sodium; K, potassium; CRP, C-reactive protein.

**Table 3:** Selected laboratory data from the entire group of patients with chronic heart failure prior to and 48 hours following CRT implantation (*n* = 64).

Variable	Before CRT median (lower-upper quartile)	48 hours after CRT median (lower-upper quartile)	*R*	*p* ^*#*^
sCR (mg/dL)	1.11 (0.925–1.35)	1,22 (0.95–1.95)	0.78	0.1846
eGFR _CKD-EPI_ (ml/min/1,73 m^2^)	65 (50.5–79)	62 (50–78)	0.78	0.1341
uAlb (mg/dL)	16.89 (6.42–55.7)	11.305 (5.24–32.61)	0.62	0.0096
uACR (mg/g)	18.37 (6.44–68.6)	10.52 (5.81–32.66)	0.63	0.0492
uNGAL (ng/mL)	14.75 (8.8–32)	14.25 (7.7–32.3)	0.48	0.65
uNCR	15.11 (8.57–29.38)	14.47 (9.73–28.245)	0.55	0.0753
uCr (mg/dL)	0.998 (0.546–1.594)	1.262 (0.614–1.749)	0.40	0.2617
Hb (g/dL)	13.4 (12.2–14.6)	13.2 (12.3–14.4)	0.87	0.0034
HCT (%)	39.8 (38.1–43.5)	39.1 (36.6–42.6)	0.82	0.0333
RBC, millions per K/*µ*L	4.47 (4.14–4.83)	4.39 (4.09–4.83)	0.79	0.0286
WBC, thousands per K/*µ*L	6.6 (5.45–8.85)	7.08 (6.24–8.85)	0.76	0.0758
PK, thousands per K/*µ*L	196 (160–238)	172 (145–221)	0.90	<0.0001
Na (mmol/L)	136.5 (134–139)	136 (134–138)	0.67	0.2343
K (mmol/L)	4.4 (4.1–4.7)	4.1 (3.9–4.5)	0.31	0.0002
CRP (mg/L)	3.65 (1.7–8.4)	14,9 (10.5––25)	0,71	0.1798

^#^Student's *t*-tests for dependent pairs to compare the significance of the difference in levels of a given parameter between points 0 and 48. Abbreviations: sCr-creatinine serum; eGFR CKD-EPI, estimated glomerular filtration rate calculated using Chronic Kidney Disease Epidemiology Collaboration; uAlb, urinary albumin concentration in a single morning sample of urine; uACR, urinary albumin to creatinine ratio; uNGAL, lipocalin associated with neutrophil gelatinase in a single morning sample of urine; uNCR, urinary NGAL to creatinine ratio; uCr, urea creatinine; Hb, hemoglobin; HCT, hematocrit; RBC, red blood cell count; WBC, white blood cell count; PK, platelet count; Na, sodium; K, potassium; CRP, C-reactive protein.

**Table 4:** Dependencies in selected laboratory data in patients with chronic heart failure 48 hours following CRT implantation depending on the NYHA class.

Variable	NYHA II (*n* = 27)		NYHA III/IV (*n* = 37)	
	*R*	*p* ^#^	*R*	*p* ^#^
sCR (mg/dL)	0.9	0.470	0.6	0.239
eGFR _CKD-EPI_ (ml/min/1.73 m^2^)	0.9	0.575	0.7	0.439
uAlb (mg/dL)	0.9	0.345	0.6	0.019
uACR (mg/g)	0.8	0.199	0.6	0.092
uNGAL (ng/mL)	0.4	0.089	0.5	0.661
uNCR (*µ*g/g)	0.4	0.636	0.5	0.083
uCr (mg/dL)	0.4	0.3	0.3	0.465
Hb (g/dL)	0.9	0.849	0.9	0.021
HCT (%)	0.8	0.379	0.8	0.003
RBC, millions per K/*µ*L	0.7	0.410	0.8	0.039
WBC, thousands per K/*µ*L	0.7	0.05	0.8	0.375
PK, thousands per K/*µ*L	0.9	<0.0001	0.9	<0.0001
Na (mmol/L)	0.7	0.410	0.7	0.3
K (mmol/L)	0.2	<0.0001	0.4	0.016

^#^Student's *t*-tests for dependent pairs. Abbreviations: sCr, creatinine serum; eGFR CKD-EPI, estimated glomerular filtration rate calculated using Chronic Kidney Disease Epidemiology Collaboration; uAlb, urinary albumin concentration in a single morning sample of urine; uACR, urinary albumin to creatinine ratio; uNGAL, lipocalin associated with neutrophil gelatinase in a single morning sample of urine; uNCR, urinary NGAL to creatinine ratio; uCr, urea creatinine; Hb, hemoglobin; HCT, hematocrit; RBC, red blood cell count; WBC, white blood cell count; PK, platelet count; Na, sodium; K, potassium.

## Data Availability

The data used to support the findings of this study are restricted by the Bioethics Committee of the University of Rzeszów in order to protect patient privacy. Data are available from Agnieszka Gala-Błądzińska (e-mail: agala.edu@gmail.com) for researchers who meet the criteria for access to confidential data.
